# Malaria in Cambodia: A Retrospective Analysis of a Changing Epidemiology 2006–2019

**DOI:** 10.3390/ijerph18041960

**Published:** 2021-02-18

**Authors:** Srean Chhim, Patrice Piola, Tambri Housen, Vincent Herbreteau, Bunkea Tol

**Affiliations:** 1 National Center for Epidemiology and Population Health, Research School of Population Health, College of Health and Medicine, Australian National University, Canberra 0200, Australia; tambrianne.housen@newcastle.edu.au; 2 Institut Pasteur du Cambodge, Phnom Penh 12201, Cambodia; ppiola@pasteur-kh.org; 3 Espace-Dev, IRD, University Antilles, University Guyane, University Montpellier, University Réunion, Phnom Penh 12201, Cambodia; vincent.herbreteau@ird.fr; 4 School of Public Health, The National Institute of Public Health, Phnom Penh 12152, Cambodia; tolbunkea@ymail.com

**Keywords:** malaria, surveillance, spatial analysis, cluster, epidemiology, GIS, trend, Cambodia

## Abstract

Background: In Cambodia, malaria persists with changing epidemiology and resistance to antimalarials. This study aimed to describe how malaria has evolved spatially from 2006 to 2019 in Cambodia. Methods: We undertook a secondary analysis of existing malaria data from all government healthcare facilities in Cambodia. The epidemiology of malaria was described by sex, age, seasonality, and species. Spatial clusters at the district level were identified with a Poisson model. Results: Overall, incidence decreased from 7.4 cases/1000 population in 2006 to 1.9 in 2019. The decrease has been drastic for females, from 6.7 to 0.6/1000. Adults aged 15–49 years had the highest malaria incidence among all age groups. The proportion of *Plasmodium (P.) falciparum* + *Mixed* among confirmed cases declined from 87.9% (*n* = 67,489) in 2006 to 16.6% (*n* = 5290) in 2019. Clusters of *P. falciparum* + *Mixed* and *P. vivax + Mixed* were detected in forested provinces along all national borders. Conclusions: There has been a noted decrease in *P. falciparum* cases in 2019, suggesting that an intensification plan should be maintained. A decline in *P. vivax* cases was also noted, although less pronounced. Interventions aimed at preventing new infections of *P. vivax* and relapses should be prioritized. All detected malaria cases should be captured by the national surveillance system to avoid misleading trends.

## 1. Introduction

Malaria, an *Anopheles* mosquito-borne disease, remains a serious public health issue globally, although it is preventable and curable [1]. According to the World Health Organization (WHO), an estimated 228 million malaria cases occurred worldwide in 2018, compared with 251 million cases in 2010 and 231 million cases in 2017 [2]. Malaria was responsible for the loss of 405,000 lives in 2018, 416,000 lives in 2017, and 585,000 lives in 2010 worldwide. Morbidity and mortality rates due to malaria vary from one global region to another. The overall malaria incidence rate was 57.4 per 1000 at-risk population, ranging from 2.6 per 1000 at-risk population in the WHO Western Pacific Region covering 37 countries of Oceania, East Asia, and Southeast Asia to 229.3 per 1000 at-risk population in the WHO African Region covering 47 countries in Africa in 2018 [2,3,4]. The deaths due to malaria were lowest at 0.4 per 100,000 population in the WHO Region of the Americas and highest at 41.0 per 100,000 in the WHO Africa Region. The global mortality rate was 10.2 per 100,000 population in 2018. [2,3,5]. 

Historically, Cambodia has had a high disease burden due to malaria since the 1950s [6,7]. Cambodia benefits from a tropical climate and is home to a variety of mosquito species, including the *Anopheles* species [8]. Malaria vectors mainly live in forests close to the Vietnamese, Laotian, and Thai borders [9,10]. People living or working in or near forested areas are at higher risk of contracting malaria [11,12]. 

Cambodia has been widely recognized as successful in the fight against malaria because of the dramatic drop in malaria cases from a peak of over 100,000 cases or 7.4 per 1000 population in 2006 to over 62,000 cases or 3.9 per 1000 population in 2018. Deaths due to malaria also dropped from around 400 reported deaths in 2006 to zero reported malaria deaths in 2017 and 2018 [13,14]. However, Cambodia still has the highest malaria burden compared to its neighboring countries—Vietnam, Thailand, and Laos—and the WHO Western Pacific Region overall [2]. The decline in malaria morbidity and mortality led the Ministry of Health to acknowledge the impact of malaria interventions combined with prevention, economic growth, improved infrastructure, and strengthening of the health system [13], all of which have enabled universal access to timely malaria diagnosis and treatment [13]. 

Built on this successful experience, since the year 2011, the government has worked with local and international partners on an elimination strategy [6,7]. Implementations were guided by the “National Strategic Plan for Elimination of Malaria in the Kingdom of Cambodia, 2011–2025” and the revised “Cambodia Malaria Elimination Action Framework, 2016–2020”. Cambodia has divided the country’s malaria-endemic areas into four zones based on the annual parasite index and malarial multi-drug resistance status [6,7]. All zones rely on quality diagnosis and treatment at health facilities, village malaria workers (VMWs), and vector control by promoting malaria prevention education and distribution of long-lasting insecticide nets [6,7]. However, to be more efficient and cost-effective, specific interventions were added for each zone [6,7]. First, the elimination zone focused on “transmission interruption” through household testing, vector control, and the expansion of VMWs [6,7]. Second, the pre-elimination zone focused on universal access to diagnosis and treatment by expanding VMWs [6,7]. Third, the reduction zone focused on the intensification plan—re-activating and scaling up VMWs, switching to new antimalarial drugs (dihydroartemisinin–piperaquine (DHA-PIP) to artesunate–mefloquine (AS-MQ)), and creating mobile malaria workers (MMWs) dedicated to the diagnosis and treatment of malaria among forest goers [7]. Finally, the non-endemic zone, defined as areas where no local transmission had been reported, uses government-run healthcare facilities’ basic services for diagnosis and treatment [6,7]. 

As part of the elimination plan, the Ministry of Health licensed the private sector to test and treat malaria patients between 2011 and 2018 to increase early diagnosis and treatment accessibility. This initiative was known as “Private Public Mix (PPM)” [7]. PPM provided private point-of-care, including private health facilities, pharmacies, and other retailers with malaria test kits and antimalarial drugs free of charge or with a subsidy [7,15,16]. Private point-of-care sites charged patients for their service provision [6,7,13]. These were rapidly scaled up in all endemic areas [15]. According to available data through malaria outlet surveys between 2011 and 2015, private point-of-care sites detected over 50% of malaria patients in the country [15]. PPM was later banned from implementing malaria testing and treatment in April 2018 [7,15]. The reason for the ban was due to malarial drug resistance concerns and the inflexibility experienced in changing testing and treatment policy in the private sector compared to government-run healthcare facilities [7]. 

The road to success has been fraught with challenges. One main challenge has been antimalarial drug resistance [17,18,19,20,21,22]. Like other countries in the Greater Mekong sub-region—China, Myanmar, Thailand, Laos, and Vietnam—Cambodia is facing the continual threat of antimalarial drug resistance [17,18,19,20,21,22,23. Cambodia first detected parasite resistance to artemisinin in 2006, but retrospective analysis of molecular markers suggested the resistance may have occurred as early as 2001 [6,7,23,24,25]. After nine years of usage, the first-line treatment—AS-MQ—was replaced by DHA-PPQ in Pailin province, in 2008, and nationally in 2010 [6,7,26]. However, the efficacy of DHA-PPQ dropped after a short period of usage [19,27,28,29]. In 2014, the cure rate of DHA-PPQ ranged from 37.5% in Siem Reap province to 89.9% in Pursat province [6,7,28]. In consultation with its partners, the Ministry of Health decided to re-introduce AS-MQ as the first-line treatment nationally in 2016 [7]. However, full coverage was delayed until 2018 due to procurement issues and funding gaps [7,30]. Cambodia relies heavily on external funding. About 70% of malaria control funding was from the Global Fund, while the remaining 30% was from a combination of government funds and other partners [6,7]. Core activities were severely affected during an external funding interruption in 2015–2016, with all activities supported by the Global Fund being suspended for more than a year. This likely influenced the increase in malaria notifications that followed in 2017 and 2018 [2,14].

In the global fight against malaria, the WHO, in the global technical strategy for malaria 2016–2030, has called for the use of surveillance systems as a core intervention in all malaria-endemic settings [31]. Data from the malaria surveillance systems are crucial to guide interventions, planning, and resource allocation [32,33,34,35,36,37,38,39,40,41]. In 2019, malaria cases in Cambodia were reported through two surveillance systems under the Ministry of Health’s umbrella. Those two systems are the Health Management Information System (HMIS) [42] and the Malaria Information System [43]. Created in 1993, with multiple enhancements, HMIS captures data from all health services, including out-patient and in-patient services at government-run healthcare facilities [42]. Specifically for malaria, a more reliable HMIS was developed in 2004. Based on these data, this study aimed to describe how malaria incidence has evolved spatially and how the sequence of untoward events and malaria control interventions may have affected geospatial trends.

## 2. Materials and Methods 

We undertook a secondary data analysis of malaria data from all government healthcare facilities in Cambodia between 2006 and 2019. Data from private providers are not captured in this dataset.

### 2.1. Data Sources

#### 2.1.1. Malaria Data 

The Epidemiology Unit of the National Centre for Parasitology, Entomology and Malaria Control provided HMIS malaria data for the cases recorded between 1 January 2006 and 31 December 2019. These aggregated data included monthly malaria cases categorized by age group, gender, type of diagnosis (rapid diagnostic test (RDT), microscope), species (*Plasmodium (P.) falciparum*, *P. vivax*, and *Mixed* meaning having both *P. falciparum* and *P. vivax*), points of care for diagnosis (public hospitals, health centers, and VMWs), case classification (uncomplicated, severe, death [44]), and hospitalization (yes, no).

HMIS is under the supervision of the Ministry of Health’s Department of Planning and Health Information. It is the largest health information system in Cambodia, collecting information from all government-run services, including out-patient, in-patient, and national program data. At the hospital level, where computers are available, data are collected and entered by hospital staff. However, at the health center or health post level, data are recorded in patients’ logbooks. Health center data are merged with data from health posts and VMWs by health center staff and aggregated using a standardized line-listing form before monthly submission to the operational district. Operational districts are responsible for entering data in the HMIS system, which is automatically synchronized to the national database of the Department of Planning and Health Information. The operational districts were created by the Ministry of Health to provide a local level to Cambodia’s health system administration (central/national level, provincial level, and operational district level) [42]. One operational district manages health facilities in one or more administrative districts and covers populations between 100,000 and 200,000 [42]. Of note, HMIS did not record malaria cases diagnosed by the private sector.

#### 2.1.2. Population Data and Maps

The population data, disaggregated by gender and age group at the district level, were obtained from an open-access dataset provided by the United Nations for Coordination of Humanitarian Affairs (OCHA) [45]. Population data were only available for 2016 [45]. To obtain population data for other years, we projected backward and forward using the National Institute of Statistics’ national population growth rate [46]. We also used the district map of Cambodia from OCHA [45].

#### 2.1.3. Untoward Events

We gathered untoward events to contextualize the malaria surveillance data. Information such as treatment failures, key interventions, and events that may have impacted the case detection were obtained from the gray literature review. We mostly relied on governmental documents, including strategic plans and strategic plan reviews for additional untoward events presented in this paper.

### 2.2. Inclusion Criteria and Setting

All government-run healthcare facilities (hospitals and health centers) reporting to the HMIS were included in this study. The number of healthcare facilities increased from 1183 in 2006 to 1350 in 2019 (Table 1). During this period, some health centers were upgraded to referral hospitals.

These health facilities are located in 24 provinces and the capital city of Phnom Penh. This includes 197 administrative districts and a total of 94 operational districts. A health center covers a catchment area of between 10,000 to 20,000 people [42].

### 2.3. Case Definition 

According to the National Treatment Guideline in Cambodia, confirmed malaria cases were persons who tested positive for *P. falciparum, P. vivax,* or both (“*Mixed*”) by RDT or microscopy [47]. 

Before 2014, unconfirmed malaria cases were also counted in the HMIS system. The definition of an unconfirmed case was a person who had not been tested for malaria with a diagnosis based only on signs and symptoms. Unconfirmed cases were counted if they:

− had fever, chills, or sweats or two of the following: headache, nausea, vomiting, diarrhea, AND
− any of the following: traveled to the forest in the previous month, had confirmed malaria in the past 28 days, traveled to a malaria-endemic area from a non-endemic area, or lived or worked around others with a recently confirmed malaria diagnosis [47].

Our analysis included all cases (confirmed and unconfirmed) for overall incidence rates and rates by sex, age, district, and seasonality. We, however, used only confirmed cases to disaggregate by *Plasmodium* species as it was not possible to identify the species among unconfirmed cases.

### 2.4. Data Analysis

We calculated the annual malaria incidence (disaggregated by species) at the national and district levels by dividing each year’s confirmed cases by their respective populations and obtaining incidences by gender and age group.

We investigated the existence of clusters [48,49] by testing two hypotheses: (1) *H*_0_ = *malaria cases in each district throughout the country are proportional to the population*, and (2) *H_A_ = malaria cases in one or more districts are statistically higher than expected proportional to the population*. We ran the cluster analysis using a spatial Poisson model with SaTScan™ version 9.6 (Information Management Services Inc., Calverton, MD, USA) at the administrative district scale, using annual malaria cases and population data [48,49]. We introduced the assumption that the cluster’s radius had to be smaller than 100 km or cover less than 50% of the country’s total malaria cases to mitigate the effect of large cluster sizes on the analysis. Such clusters were split into smaller clusters. Another assumption we introduced was that the relative risk had to be greater than three. This restriction allowed us to focus the analysis on high-burden clusters. The default of Monte Carlo replication of 999 was used with a cut-off *p*-value < 0.001 [48,49]. In this publication, we use the term “cluster” in reference to SaTScan’s “most likely cluster”, which are clusters detected during the first run. In the Poisson model, after the first run, SaTScan removes all data inside the detected clusters and treats them as “no location”, “no case”, “no population”. It then runs again to detect clusters with the remaining data. These newly detected clusters are called “secondary clusters”. Since we are only interested in high-burden areas or “most likely clusters”, we did not allow the option to display “secondary clusters” in our analysis result.

We used Stata version 15.1 (StataCorp, College Station, TX, USA) for descriptive data analysis and R version 4.0 (R Foundation for Statistical Computing, Vienna, Austria) to produce graphics and maps.

## 3. Results

### 3.1. Overall Malaria Cases and Incidence Rate 

**Figure 1:**
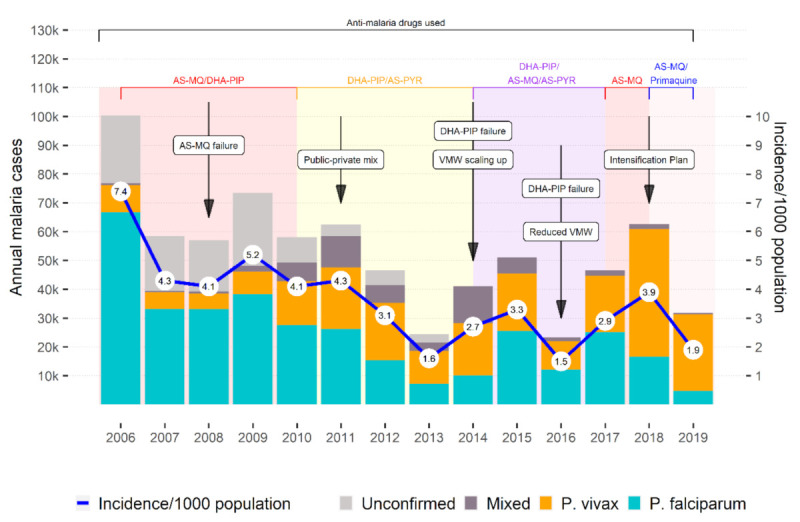
Number of confirmed (by species) and unconfirmed malaria cases, incidence (confirmed and unconfirmed) per 1000 population, and untoward events, in 2006–2019, Cambodia. Abbreviations: AS-MQ, artesunate–mefloquine; DHA-PIP, dihydroartemisinin–piperaquine; AS-PYR, artesunate–pyronaridine; VMW, village malaria worker.

A total of 737,210 malaria cases (confirmed or unconfirmed) was reported to HMIS between 2006 and 2019 (3.6/1000 population) (Figure 1). The highest incidence was reported in 2006, with 7.4 cases/1000 population (100,322 cases), and the lowest incidence was reported in 2016, with 1.5 cases/1000 (23,367 cases). However, the incidence did not decrease regularly over the time period, with a sharp increase to 3.9 cases/1000 in 2018 (62,582 cases) followed by a drop to 1.9 in 2019 (32,597 cases). Between 2006 and 2013, unconfirmed malaria cases were included in the HMIS. Unconfirmed cases made up a large proportion of all reported cases between 2006 and 2010, and subsequently dropped between 2011 and 2013. Nevertheless, the number of confirmed cases was particularly high in 2006, and stable between 2007 and 2011. Between 2007 and 2011, the total number of malaria cases was quite stable, with a drop in unconfirmed cases being compensated by an increase in the confirmed cases. Since 2014, all reported malaria cases have met the confirmed case definition. 

After the Private Public Mix (PPM) program was initiated in 2011, the malaria incidence trends (in which data from the private sector were not included) dropped dramatically from 4.3 cases/1000 in 2011 to 1.6 cases/1000 in 2013. As a significant proportion of malaria cases were detected by private sector during this period, the decreasing trend in incidence is unlikely to represent the actual incidence of malaria in Cambodia for this period of time. 

Therefore, data represented in this study must be considered in light of the changes in the surveillance case definition over time and changes in reporting sources.

The proportion of *P. falciparum + Mixed* peaked at 87.9% (*n* = 67,489) of all confirmed malaria cases recorded in 2006 to reach the lowest rate of 16.6% (*n* = 5290) of all recorded cases in 2019. The proportion of *P. falciparum + Mixed* was highest between 2006 and 2009, when the country was using *P. falciparum*-only RDTs. The proportion of *P. vivax + Mixed* exceeded the proportion of *P. falciparum + Mixed* between 2011 and 2014, and then again from 2017 onwards. The change occurred a year after Cambodia started using dual RDT in late 2009, which detected both *P. falciparum* and *P. vivax.* More detailed information can be found in the Supplementary Materials (Figure S1). 

### 3.2. Incidence by Sex 

**Figure 2:**
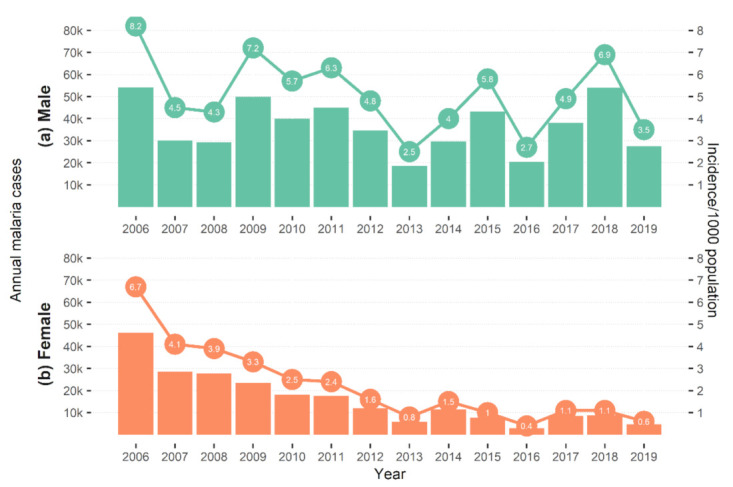
Malaria incidence (confirmed + unconfirmed cases) per 1000 males (**a**) and females (**b**), in 2006–2019, Cambodia.

The incidence of malaria (confirmed + unconfirmed cases per 1000 population) among women decreased steadily from 6.7/1000 in 2006 to 0.5/1000 in 2019 (average decrease of 4.6% per year since 2006). This was not observed for men, whose incidence fluctuated widely between a maximum of 8.2/1000 in 2006 and a minimum of 2.5/1000 in 2013 (Figure 2). Malaria incidence among males was consistently higher throughout the analysis period.

### 3.3. Incidence by Age Group

**Figure 3:**
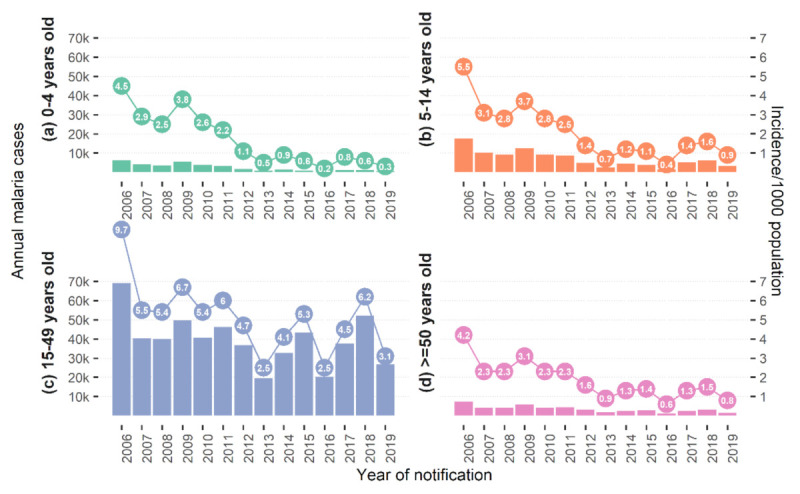
Here: Malaria incidence (confirmed + unconfirmed cases) per 1000 by age groups (**a**) 0–4, (**b**) 5–14, (**c**) 15–49, (**d**) ≥50, in 2006–2019, Cambodia.

Adults aged 15–49 years had the highest malaria incidence (confirmed + unconfirmed) among all age groups. The incidence in this age group decreased from 9.7 cases/1000 in 2006 to 3.1/1000 in 2019 and was lowest at 2.5 in 2013 and 2016 (Figure 3). In other age groups (under five years, 5–14 years, 49 years and above), malaria incidence rates presented similar trends, albeit much lower rates.

### 3.4. Seasonality 

**Figure 4:**
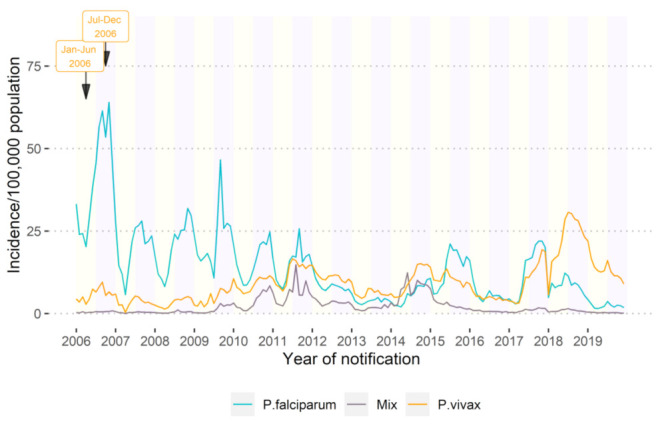
Malaria incidence (confirmed + unconfirmed) per 100,000 populations per month (2006–2019), in 2006–2019, Cambodia.

Both *P. falciparum + Mixed* and *P. vivax + Mixed* presented a similar seasonal pattern over time, except in 2016 and 2019 (Figure 4). Fewer *P. falciparum + Mixed* and *P. vivax + Mixed* cases were recorded between February and May, while the high malaria season was observed between June and January. A more detailed visualization of cases by month is available in the Supplementary Materials (Figure S2). 

### 3.5. Geographical Distribution 

#### 3.5.1. Clusters of *P. falciparum* + *Mixed*

**Figure 5:**
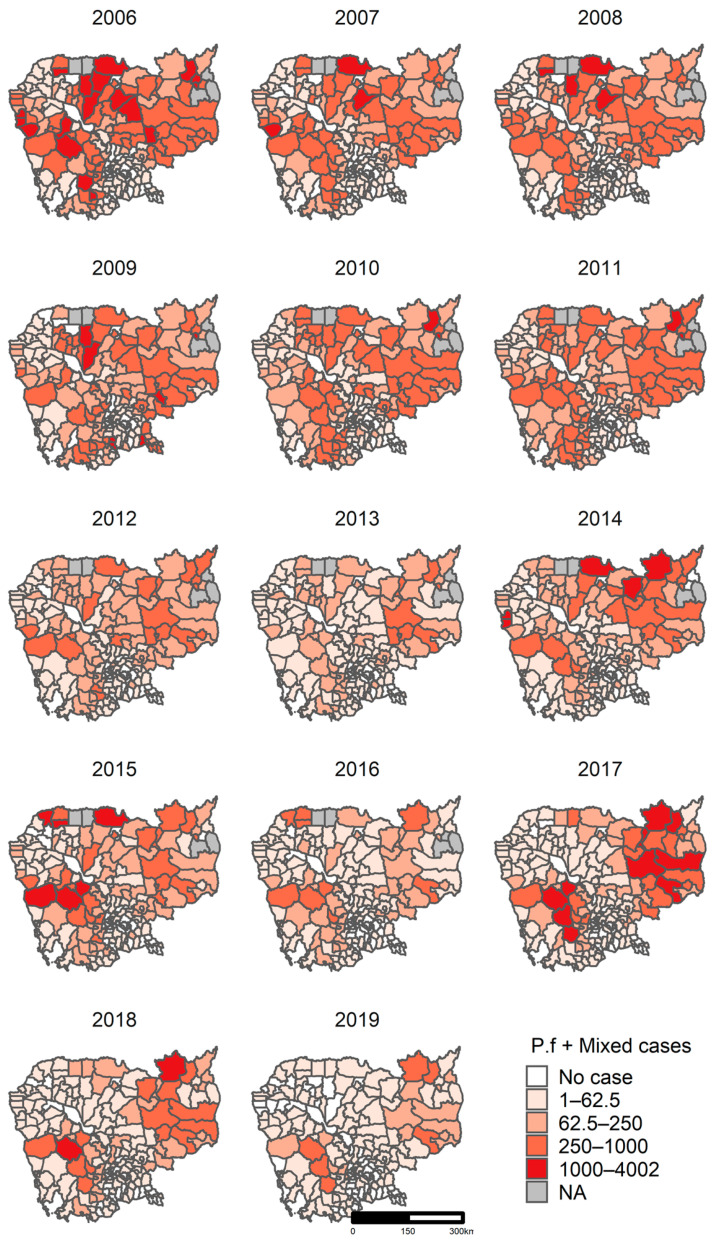
Confirmed *P. falciparum + Mixed* case distribution by district, 2006–2019, Cambodia. Abbreviation: NA, not applicable (no data).

The number of confirmed *P. falciparum* + *Mixed* cases ranged from zero to 4002 by district per year (Figure 5). The districts with a high burden of malaria, over 1000 cases per year, were seen along the national borders, in the western provinces (Pursat, Kampong Speu, Koh Kong, and Pailin), northern provinces (Preah Vihear and Udor Mean Chey), northeastern provinces (Kratie, Stung Treng), and eastern provinces (Mondul Kiri and Ratanakiri) (Figure 5). The majority of districts with a low malaria burden, lower than 250 cases per year, were observed in central and southern provinces (Figure 5).

**Figure 6:**
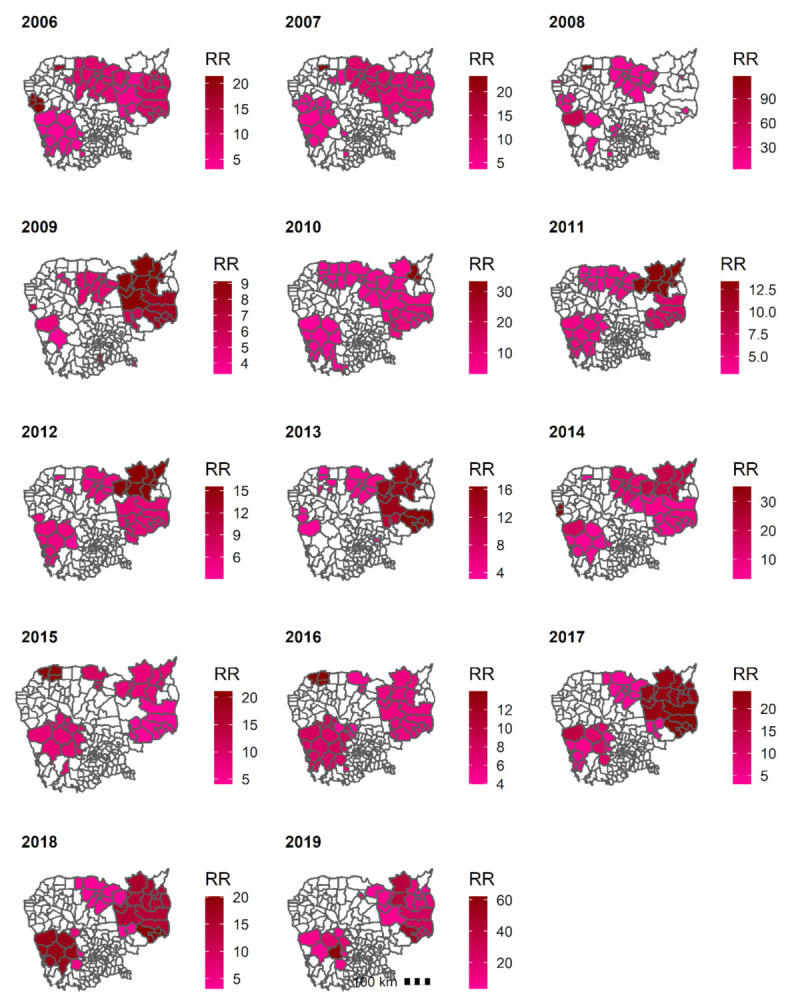
Clusters of confirmed *P. falciparum + Mixed* cases, 2006–2019, Cambodia. Abbreviation: RR, relative risk.

The cluster analysis revealed a decrease in the size and number of the clusters of *P. falciparum + Mixed* cases during the last three years, 2017, 2018, and 2019 (Figure 6). Between 2017 and 2019, these clusters were detected in districts within the northeastern and eastern provinces (Kratie, Stung Treng, Mondul Kiri, and Ratanak Kiri) and districts within the western provinces (Kampong Speu and Pursat). 

#### 3.5.2. Clusters of *P. vivax + Mixed*

**Figure 7:**
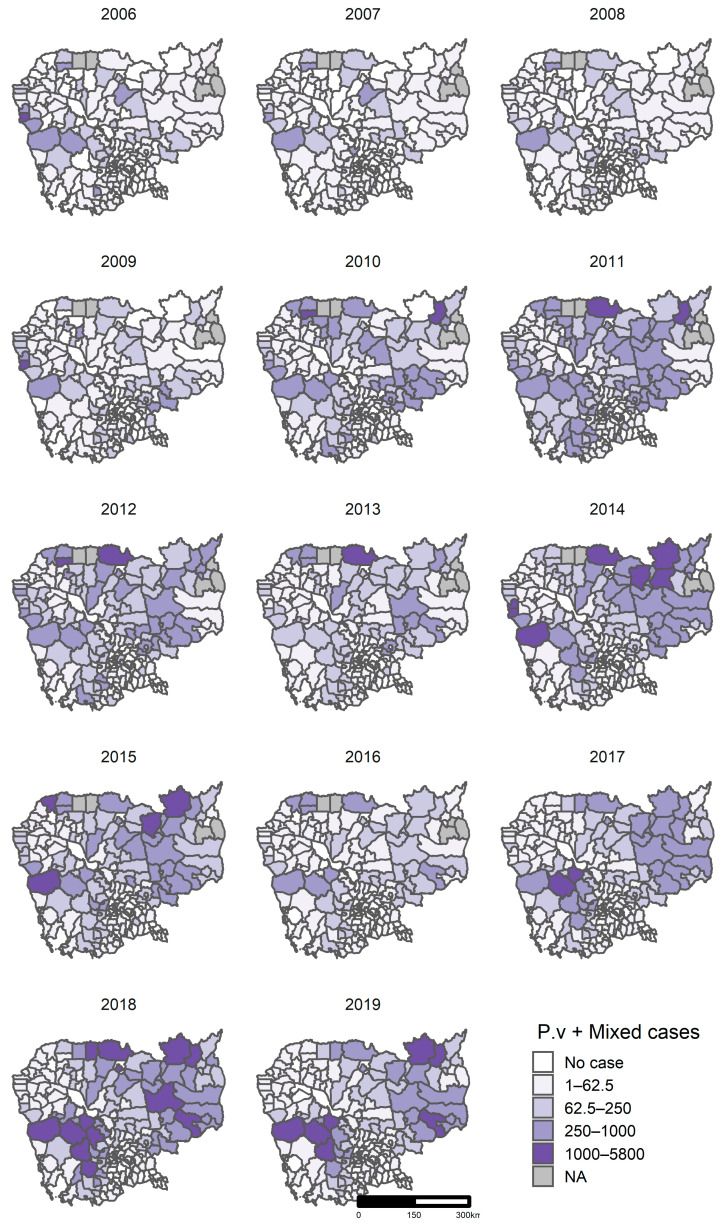
Confirmed *P. vivax + Mixed* cases by district, 2006–2019, Cambodia. Abbreviation: NA, not applicable.

The number of confirmed *P. vivax* + *Mixed* cases ranged from zero to 5800 cases by district per year. As for *P. falciparum* + *Mixed*, the districts with a high burden of malaria, over 1000 cases per year, were seen along the national borders, in the western provinces (Pursat, Kampong Speu, Koh Kong, and Pailin), the northern provinces (Preah Vihear and Udor Mean Chey), northeastern provinces (Kratie, and Stung Treng), and the eastern provinces (Mondul Kiri and Ratanak Kiri) (Figure 7). 

**Figure 8:**
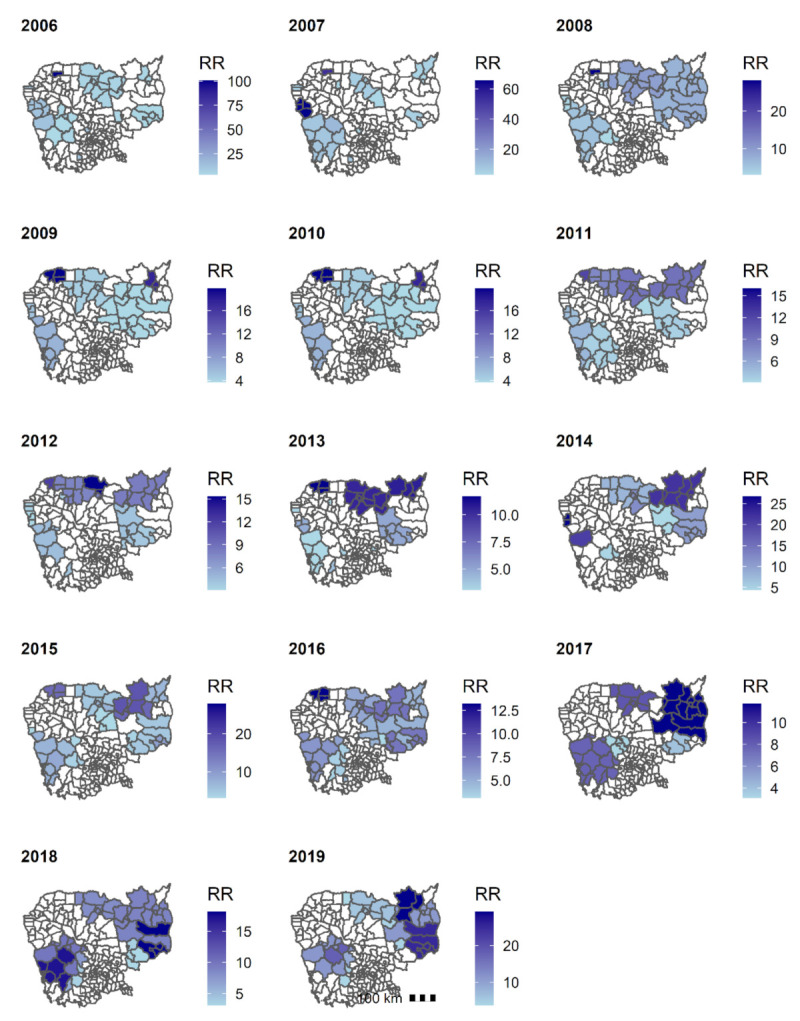
Clusters of confirmed *P. vivax + Mixed* cases, 2006–2019, Cambodia. Abbreviation: RR, relative risk.

As for *P. falciparum,* fewer and smaller clusters of *P. vivax* + *Mixed* cases were detected in the last three years (2017–2019) (Figure 8). Between 2017 and 2019, clusters were also seen along the national borders, in the northern provinces (Preah Vihear and Udor Mean Chey), northeastern provinces (Kratie, and Stung Treng), eastern provinces (Mondul Kiri and Ratanak Kiri), and the western provinces (Pursat, Kampong Speu, and Koh Kong). 

## 4. Discussion 

This study describes how malaria has evolved spatially from 2006 to 2019 in Cambodia. The peak malaria seasons were found to be between June and January. A similar finding was described by Maude et al. in 2014, based on Cambodia’s national malaria data between 2004 and 2013 [50]. The rainy season starts one month before the malaria season commences (May vs. June), while it ends two months before the malaria season ends (November vs. January). Previous studies in China, Tibet, and Niger found that rainfall contributed to increased *Anopheles* and malaria incidence [51,52,53,54]. 

Our analysis detected *P. falciparum + Mixed* and *P. vivax + Mixed* clusters in seven provinces along national borders with Vietnam, Laos, and Thailand. These areas are covered by evergreen broadleaf forests [55] where *P. falciparum*- and *P. vivax*-infected *Anopheles* are prevalent [8]. This geographical distribution of malaria in Cambodia was previously known [50]. However, this analysis provides updated information confirming that these areas, in the elimination phase, are the top priority. The population’s livelihood in the greater incidence regions remains dependent on the forests, including logging precious woods, agriculture activities, and residency on the fringe of the forests [11]. To reduce *P. falciparum* and *P. vivax*, the intensification plan, which was effective in 2019, should be maintained, and innovative approaches aimed at blocking transmission from forests to communities should be considered. The main limitation of this cluster analysis is that we can only locate cases at the place of diagnosis, i.e., the location of health facilities, and it is not possible to know where the case contracted malaria. This is a common limitation when using surveillance data.

The overall decrease in malaria has been observed mainly in women, while the annual incidence has fluctuated in men, with the highest rate observed among men aged 15–49 years old. Although the number of cases was similar in men and women in 2006 (8.2 vs. 6.7/1000 population), the gap increased considerably in 2019 (3.5 vs. 0.6/1000 population). The number of cases in women represented only 14% of the total cases. This notable difference in malaria incidence in males and females is likely related to a change in exposure. The evolution of women’s daily activities may have become less related to forests, with adult males being the most exposed to forest activities and, therefore, most at risk of malaria infections [11,12,56]. According to malaria surveys of forest goers, 72.2% in 2004, 70.7% in 2007, 69.9% in 2010, 78.8% in 2013, and 85.3% in 2017 were males aged 15 years old or older while 17.1% in 2004, 20.7% in 2007, 20.6% in 2010, 15.6% in 2013, and 9.7% in 2017 were females aged 15 years old or older [57,58,59,60]. From these figures, the proportion of females who go to forests has decreased by half since 2010. Another possible reason could be the increasing distance between villages and forests due to deforestation. In Cambodia, two primary vectors—*Anopheles dirus* and *Anopheles minimus*—play an important role in spreading malaria [61]. *Anopheles dirus* is more efficient (higher percentage of mosquitoes tested positive for *P. falciparum* and *P. vivax*) than *Anopheles minimus* [61]. They also have different habitats, with *Anopheles dirus* living in natural forests and forest fringes, and *Anopheles minimus* living around rice fields and forest fringes [61,62]. With Cambodia undergoing rapid deforestation in recent years [62], the territory of *Anopheles dirus* could decrease, leading to a lower risk of malaria transmission. However, deforestation activities correspond to an influx of loggers into *Anopheles dirus* habitat which can lead to increases in malaria cases. This highlights the importance of interventions targeting forest goers to block malaria transmission in forests.

Several key factors may have impacted the malaria trends, although our study design does not provide evidence to prove causality. 

First, we observed that all the upward trends in malaria have coincided with periods of high treatment failure rates for *P. falciparum*, while all of the observed declines in incidence occurred one to two years after a change in first-line treatment. It is plausible that the delayed parasite clearance due to ineffective antimalarial drugs increased malaria transmission among high-risk populations. In 2009, the total malaria cases increased by 29% (*n* = 16,493) compared to 2008. This rebound occurred after the first-line antimalarial—AS-MQ—showed high treatment failure rates [7,24,25,61,62,63,64,65,66,67,68,69,70,71]. A new first-line antimalarial (DHA-PIP) was introduced nationally in 2010 to replace AS-MQ [7,15,72]. This was followed by an observed reduction of *P. falciparum* cases in Cambodia until 2013. DHA-PIP treatment failures emerged after three years of usage [27,72,73]. Total malaria cases increased again by 68% (*n* = 16,679) in 2014 compared to 2013. Malaria notifications continued to increase in Cambodia until 2018. Of note, the ineffective DHA-PIP was still used in several parts of Cambodia due to procurement challenges until 2018 [7,30]. This finding suggests that antimalarial drug efficacy is key to controlling *P. falciparum*. Timely surveillance of drug efficacy provides critical information to respond to a malaria epidemics promptly. 

Another key factor was that the dramatic drop in malaria cases was likely associated with the changing use of the private sector and an important limitation of the HMIS that did not capture data from the private sector. The private sector played a significant role in changing the national malaria trends between 2011 and 2018. However, the most substantial impact would have occurred between 2011 and 2013, when malaria incidence dropped from 4.1 to 1.6/1000 population with a sharper decrease in *P. falciparum* notifications compared to *P. vivax*. This drop was possibly due to the Private Public Mix (PPM) project piloted in 2011 and scaled up in 2012 [7,15,16]. It may have favored larger access to early detection and treatment of infections since more than 50% of antimalarial drugs were delivered at private points of care between 2011 and 2013 [16,73]. This is consistent with the findings from national malaria surveys that showed 7.3% of malaria patients used the private sector in 2007, 41.1% in 2010, and 56.4% in 2013, with a reduction to 30.3% in 2017 [57,58,59,60]. Therefore, the decreasing trend of malaria cases recorded in the HMIS between 2011 and 2013 could be related to the increasing use of the private sector, which was not included in the HMIS before it was banned in April 2018. This highlights the importance of comprehensive surveillance with mandatory reporting requirements for public and private sectors to the HMIS to avoid undercounting or misleading trends.

The decreasing trends may have been overestimated due to the inclusion of unconfirmed cases between 2006 and 2013, when the malaria test was not available throughout the country. Unconfirmed cases may include non-malaria cases with similar clinical symptoms. The malaria cases were overestimated if the unconfirmed cases were included and underestimated if the unconfirmed cases were excluded. However, there are no data on the proportion of actual cases among the unconfirmed cases. Changing case definitions and diagnostics has a significant impact on surveillance data and therefore it is important to recognize such events when interpreting surveillance data.

The role of VMWs also played a key role in malaria trends in Cambodia. The malaria incidence increased from 2.7/1000 population in 2014 to 3.3/1000 population in 2015. This increase happened after the scaling up of VMWs in 2014 [7,15,74]. VMWs provided free services in villages and reported to the HMIS through health centers, leading to greater malaria notifications. VMWs administered 41% of all antimalarial drugs in 2015 [73]. The scaling up of VMWs helped change health-seeking behaviors with a noted decrease in private sector services and improved HMIS sensitivity.

In contrast, malaria cases dropped to the lowest point in 2016 but increased again in 2017 and 2018. This occurred after many VMWs were laid off, and all other activities supported by Global Fund funding were suspended between July 2015 and December 2016 [30,75]. The immediate drop in malaria cases in 2016 may be due to the absence of notifications from VMWs. This finding implied that early access to diagnosis and treatment through VMWs or care points is another critical feature to control and eliminate malaria. Funding to maintain VMWs, or other equivalent interventions, should be prioritized until elimination is confirmed.

One more key factor was the intensification plan initiated in 2018 to respond to increasing case notifications in 2017 and 2018. A significant drop in malaria cases from 3.9/1000 in 2018 to 1.9/1000 in 2019 was observed after the plan started. For the first time, both species—*P. falciparum* and *P. vivax*—had a dramatic decline. The success of the new intensification plan targeting forest goers provides sufficient evidence that this plan should be maintained.

The final key factor, nationwide crackdowns by police and forest rangers on illegal logging, may also have had an impact on malaria trends. The massive crackdown started in 2019. It prevented the highest risk groups (forest goers) from entering forests where most malaria transmission was occurring [74]. It is of interest that the effectiveness of malaria control programs may be positively affected by external and independent factors such as anti-deforestation activities. 

Concerning the *Plasmodium* species, an increase in the proportion of *P. vivax* was observed over time. Two reasons may have contributed to these changes. Firstly, the index of species was likely affected by the shift from *P. falciparum-only* RDT to dual RDT, which could have detected both *P. falciparum* and *P. vivax*. As previously shown, *P. falciparum* notifications were predominant before 2010. This was due to Cambodia using *P. falciparum*-only RDT before 2010 [76]. In that period, *P. vivax* cases could only be detected using microscopy, for which availability was limited [77]. According to an outlet survey in 2015, microscopy was available in only 28% of government health facilities included in the study. The dual RDT was only supplied in late 2009 [76]. One year after using the dual RDT, the proportion of *P. vivax* exceeded the proportion of *P. falciparum*. However, *P. vivax* possibly remains under-detected due to the RDT’s low sensitivity (ability to detect true positives) to *P. vivax* [78]. RDTs do not perform well in individuals with low parasitemia (<100 parasites/μL) [78]. According to a meta-analysis study, the sensitivity of RDT in detecting *P. vivax* ranges from 57% to 77% compared to a polymerase chain reaction (PCR) as a gold standard [78]. Secondly, the proportion of malaria patients with *P. vivax* has increased. Relapses may explain these trends. Relapses among *P. vivax* patients were described as early as 1893 [79]. According to Taylor et al., along the Thailand–Myanmar border, neighboring countries of Cambodia, 75% of *P. vivax* patients receiving standard treatment (without primaquine radical curative treatment) relapsed within a 12-month follow-up period [80]. One *P. vivax* patient is thought to have multiple episodes of relapse, but the exact number of relapses per lifetime per person has not yet been confirmed [81]. The accumulation of *P. vivax* relapses constitutes a large proportion of all *P. vivax* cases and maintains the source of *P. vivax* infections. A radical cure for *P. vivax,* piloted in four provinces of Cambodia (Kampong Speu, Kampong Chhnang, Battambang, and Pailin), should also be prioritized in all *P. vivax* clusters. 

In contrast to *P. vivax,* the proportion of *P. falciparum* notifications declined faster over time. The decline in *P. falciparum* notifications may have resulted from multiple factors. Firstly, the enhanced *P. falciparum* elimination efforts by the Ministry of Health have likely had a key role to play, such as interventions including expanding testing and treating sites to facilitate the early diagnosis and treatment of cases [6,7,13]. In addition, a focus on the interruption of *P. falciparum* transmission, or the 1-3-7 strategy (reporting a confirmed case within one day, investigating within three days, and taking measures to prevent further transmission within seven days), in elimination areas may have also contributed to this decline [7,82,83]. Secondly, *P. falciparum* is curable. The impact from effective interventions in reducing *P. falciparum* cases should be rapidly seen. Thirdly, biologically, there is evidence that prior exposure to *P. vivax* suppresses the course of *P. falciparum* infection [84,85,86,87]. It is possible that this factor contributed to the decline in *P. falciparum*. However, the proportion by which *P. falciparum* is reduced by prior exposure to *P. vivax* is not known. 

The overall *P. falciparum* prevalence decrease was concurrent with the emergence of drug resistance in *P. falciparum*. The scaling up of improved access to early testing and treatment probably had a greater impact than the emergence of drug resistance. Ongoing surveillance of antimalarial drug resistance also ensured that first-line treatments were promptly changed to efficient regimens.

## 5. Conclusions 

In this study, we used the nationwide surveillance data collected in the last 14 years between 2006 and 2019. There was a noted decrease in notifications of *P. falciparum* in 2019, suggesting that the intensification plan should be maintained. *P. vivax* showed a slower but promising declining trend. Interventions aimed at achieving *P. falciparum* elimination and preventing new *P. vivax* infections and relapses should be prioritized. In the context of malaria elimination, all cases detected outside the national system should be reported to the national system to avoid misleading trends.

## Figures and Tables

**Table 1:** Number of health centers and hospitals by year, reporting to the Health Management Information System (HMIS), Cambodia, 2016 and 2019.

Year	Number of Health Centers	Number of Hospitals	Total Malaria Case Notifications	Population (a)
2006	1087	96	1183	13,474,489
2007	1087	96	1183	13,676,693
2008	1087	96	1183	13,880,509
2009	1087	96	1183	14,090,208
2010	1087	96	1183	14,308,740
2011	1087	96	1183	14,537,886
2012	1087	96	1183	14,776,866
2013	1087	96	1183	15,022,692
2014	1138	104	1242	15,270,790
2015	1148	107	1255	15,517,635
2016	1168	111	1279	15,762,370
2017	1195	116	1311	16,005,373
2018	1213	121	1334	16,245,454
2019	1225	125	1350	16,489,135

Population data were only available for 2016 [45]. To obtain population data for other years, we projected backward and forward using the National Institute of Statistics’ national population growth rate [46].

## Data Availability

Restrictions apply to the availability of these data. Data was obtained from the National Center for Parasitology Entomology and Malaria Control (CNM) and were made available by Bun Bunkea Tol (co-author, tolbunkea@ymail.com) with the permission of CNM.

## Supplementary Materials

The following are available online at https://www.mdpi.com/1660-4601/18/4/1960/s1, Figure S1: Proportions of malaria confirmed cases by *Plasmodium* species in Cambodia between 2006 and 2019, Figure S2: Malaria cases (confirmed + unconfirmed) by month (2006–2019), from all government healthcare facilities in Cambodia.

## Abbreviations

AS-MQ: artesunate-mefloquine, AS-PYR: artesunate-pyronaridine, DHA-PIP: dihydroartemisinin-piperaquine, HMIS: Health Management Information System, MMW: mobile malaria worker, *P. falciparum*: *Plasmodium falciparum*, *P. vivax*: *Plasmodium vivax*, VMW: village malaria worker, PPM: Private Public Mix.
