# Supplementary material for: Malaria in Cambodia: A Retrospective Analysis of a Changing Epidemiology 2006–2019
Source: Int J Environ Res Public Health. 2021 Feb 18;18(4):1960. doi: 10.3390/ijerph18041960 (PMC7922556; doi:10.3390/ijerph18041960)
Supplement: Supplementary file 1 [file ijerph-18-01960-s001.pdf]

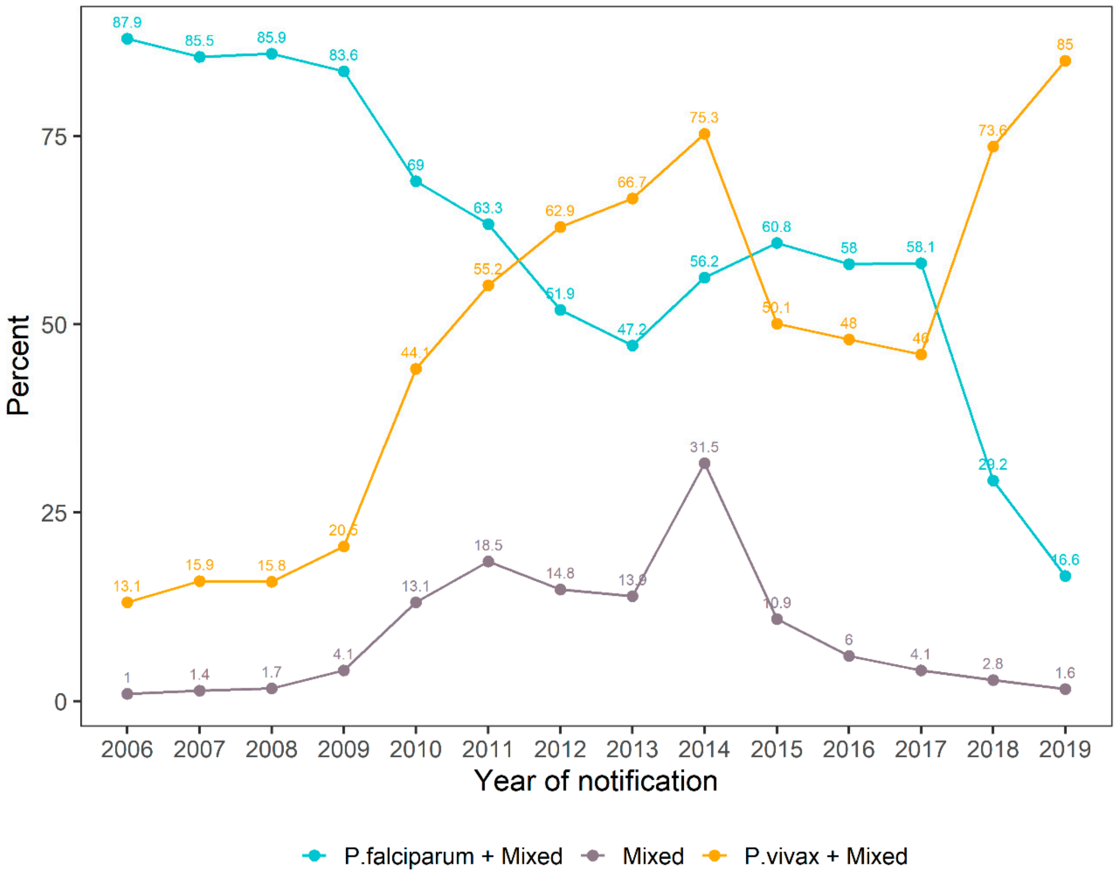

Figure S1. Proportions of malaria confirmed cases by Plasmodium species in Cambodia between 2006 and 2019.

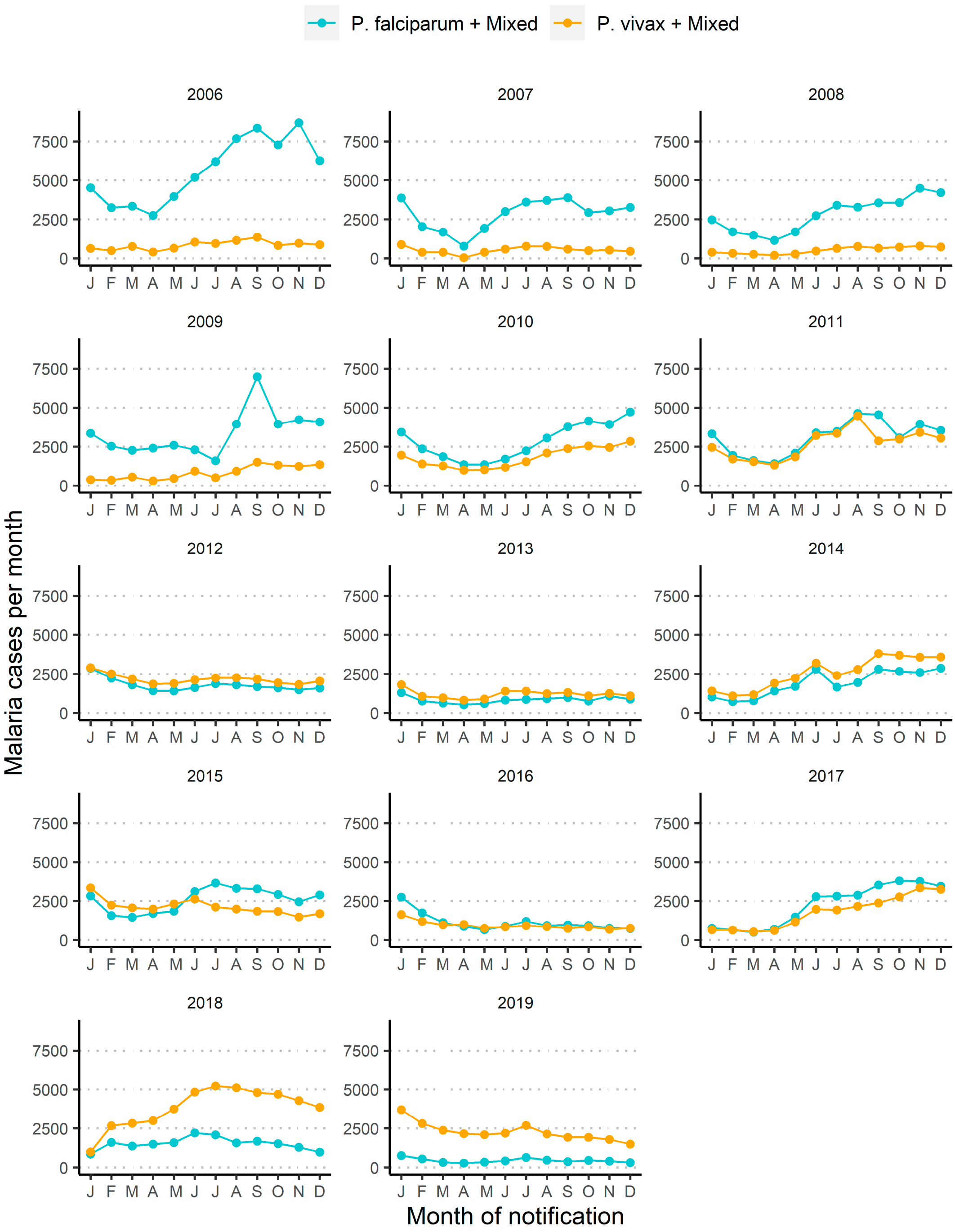

Figure S2. Malaria cases (confirmed + unconfirmed) by month (2006–2019), from all government healthcare facilities in Cambodia.
